# Supplementary figures and images for: MiRNAs Expression Profiling of Bovine (Bos taurus) Testes and Effect of bta-miR-146b on Proliferation and Apoptosis in Bovine Male Germline Stem Cells
Source: Int J Mol Sci. 2020 May 28;21(11):3846. doi: 10.3390/ijms21113846 (PMC7312616; doi:10.3390/ijms21113846)

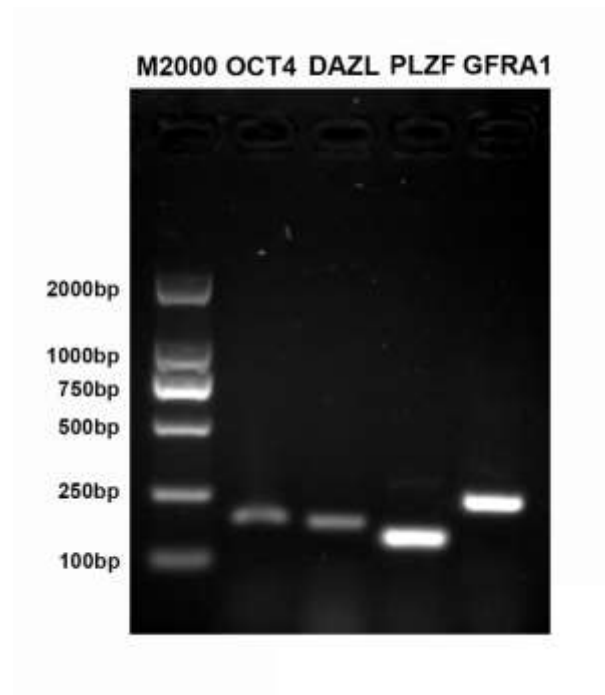

**Figure S1.** Electrophoretogram for the marker genes of bovine male germline stem cells in RT-PCR.

Supplement: Supplementary file 1 [file ijms-21-03846-s001.zip › ijms-777696-final supplementary/Figure S1.pdf]
